# Detection of SARS-CoV-2 infection in the general population by three prevailing rapid antigen tests: cross-sectional diagnostic accuracy study

**DOI:** 10.1186/s12916-022-02300-9

**Published:** 2022-02-24

**Authors:** Roderick P. Venekamp, Irene K. Veldhuijzen, Karel G. M. Moons, Wouter van den Bijllaardt, Suzan D. Pas, Esther B. Lodder, Richard Molenkamp, Zsofi Igloi, Constantijn Wijers, Claudy Oliveira dos Santos, Sylvia B. Debast, Marjan J. Bruins, Khaled Polad, Carla R. S. Nagel-Imming, Wanda G. H. Han, Janneke H. H. M. van de Wijgert, Susan van den Hof, Ewoud Schuit

**Affiliations:** 1 Julius Center for Health Sciences and Primary Care, University Medical Center Utrecht, Utrecht University, Utrecht, The Netherlands; 2 Centre for Infectious Disease Control, National Institute for Public Health and the Environment (RIVM), Bilthoven, The Netherlands; 3 Cochrane Netherlands, University Medical Center Utrecht, Utrecht University, Utrecht, The Netherlands; 4 Microvida Laboratory for Medical Microbiology, Amphia Hospital, Breda, The Netherlands; 5 Microvida Laboratory for Medical Microbiology, Bravis Hospital, Roosendaal, The Netherlands; 6 Public Health Service West-Brabant, Breda, The Netherlands; 7 Department of Viroscience, Erasmus MC, Rotterdam, The Netherlands; 8 Public Health Service Rotterdam-Rijnmond, Rotterdam, The Netherlands; 9 Laboratory of Medical Microbiology and Infectious Diseases, Isala Hospital, Zwolle, The Netherlands; 10 Public Health Service IJsselland, Zwolle, The Netherlands

**Keywords:** SARS-CoV-2, COVID-19, Rapid antigen tests, Diagnostic accuracy

## Abstract

**Background:**

Rapid antigen diagnostic tests (Ag-RDTs) are the most widely used point-of-care tests for detecting SARS-CoV-2 infection. Since the accuracy may have altered by changes in SARS-CoV-2 epidemiology, indications for testing, sampling and testing procedures, and roll-out of COVID-19 vaccination, we evaluated the performance of three prevailing SARS-CoV-2 Ag-RDTs.

**Methods:**

In this cross-sectional study, we consecutively enrolled individuals aged >16 years presenting for SARS-CoV-2 testing at three Dutch public health service COVID-19 test sites. In the first phase, participants underwent either *BD-Veritor System (Becton Dickinson*), *PanBio (Abbott*), or *SD-Biosensor (Roche Diagnostics*) testing with routine sampling procedures. In a subsequent phase, participants underwent SD-Biosensor testing with a less invasive sampling method (combined oropharyngeal-nasal [OP-N] swab). Diagnostic accuracies were assessed against molecular testing.

**Results:**

Six thousand nine hundred fifty-five of 7005 participants (99%) with results from both an Ag-RDT and a molecular reference test were analysed. SARS-CoV-2 prevalence and overall sensitivities were 13% (188/1441) and 69% (129/188, 95% CI 62–75) for BD-Veritor, 8% (173/2056) and 69% (119/173, 61–76) for PanBio, and 12% (215/1769) and 74% (160/215, 68–80) for SD-Biosensor with routine sampling and 10% (164/1689) and 75% (123/164, 68–81) for SD-Biosensor with OP-N sampling. In those symptomatic or asymptomatic at sampling, sensitivities were 72–83% and 54–56%, respectively. Above a viral load cut-off (≥5.2 log_10_ SARS-CoV-2 E-gene copies/mL), sensitivities were 86% (125/146, 79–91) for BD-Veritor, 89% (108/121, 82–94) for PanBio, and 88% (160/182, 82–92) for SD-Biosensor with routine sampling and 84% (118/141, 77–89) with OP-N sampling. Specificities were >99% for all tests in most analyses. Sixty-one per cent of false-negative Ag-RDT participants returned for testing within 14 days (median: 3 days, interquartile range 3) of whom 90% tested positive.

**Conclusions:**

Overall sensitivities of three SARS-CoV-2 Ag-RDTs were 69–75%, increasing to ≥86% above a viral load cut-off. The decreased sensitivity among asymptomatic participants and high positivity rate during follow-up in false-negative Ag-RDT participants emphasise the need for education of the public about the importance of re-testing after an initial negative Ag-RDT should symptoms develop. For SD-Biosensor, the diagnostic accuracy with OP-N and deep nasopharyngeal sampling was similar; adopting the more convenient sampling method might reduce the threshold for professional testing.

**Supplementary Information:**

The online version contains supplementary material available at 10.1186/s12916-022-02300-9.

## Background

In the first phase of the pandemic, all testing at Dutch public health service COVID-19 test sites was done with molecular tests. Molecular tests, mainly real-time reverse-transcriptase polymerase chain reaction (RT-PCR), are currently still considered reference tests for SARS-CoV-2 [1]. However, molecular testing platforms are typically only available in centralised laboratories and most of them require sample batching, thereby causing delays in delivering test results. Persons with symptoms were — and still are — strongly advised to isolate themselves until a negative test result has been obtained. Reducing test-to-result delays is therefore considered important. Point-of-care antigen tests have that potential and were introduced for testing of symptomatic persons at Dutch public health service test sites in November 2020. Later, these were also introduced for testing of asymptomatic close contacts, to gain entry to places and events where physical distancing is difficult to achieve or enforce, for travel, and for self-testing at home. Rapid lateral flow antigen diagnostic tests (Ag-RDTs) are the most promising and widely used point-of-care tests [2]. They require no or minimal equipment, provide a result within 15 min, and can be performed in a range of settings. In the current phase of the pandemic (i.e. the winter season of 2021–2022), with a new surge of SARS-CoV-2 infections occurring even in countries with high COVID-19 vaccination coverage, Ag-RDTs play a pivotal role as countries are reopening and physical distancing measures are increasingly relaxed.

Thus far, multiple studies investigated the diagnostic accuracy of SARS-CoV-2 point-of-care tests [3–5]. However, most of these studies had a limited sample size, used specimens that were left-over after molecular testing, or included symptomatic individuals only. We conducted a large diagnostic accuracy study in late 2020/early 2021 in which two SARS-CoV-2 Ag-RDTs (*BD Veritor*^*tm*^ *System by Becton Dickinson* (‘BD-Veritor’) and *Roche/SD Biosensor by Roche Diagnostics* (‘SD-Biosensor’)) were compared to RT-PCR [6]. However, we limited that evaluation to asymptomatic and presymptomatic close contacts of individuals with confirmed SARS-CoV-2 infection, and the commonly used *PanBio by Abbott* (‘PanBio’) Ag-RDT was not included in that study. Furthermore, the diagnostic accuracy of Ag-RDTs may have altered over time due to changes in SARS-CoV-2 epidemiology, indications for testing, and roll-out of COVID-19 vaccination. Diagnostic accuracy may also be impacted by sampling technique. Many Ag-RDTs require deep nasopharyngeal (NP) sampling, which is often considered to be unpleasant, whereas oropharyngeal combined with superficial nasal (OP-N) sampling might suffice.

In the first phase of the current study, we therefore evaluated the diagnostic accuracies of three Ag-RDTs (BD-Veritor, SD-Biosensor, and PanBio) that are commonly applied in Dutch test sites, using the sampling techniques that are routinely used by those test sites in individuals aged 16 or older irrespective of their indication for testing, symptomatology, and COVID-19 vaccination status. In the second phase of the study, we aimed to evaluate the diagnostic accuracies of two of the Ag-RDTs (SD-Biosensor and PanBio) when using a less invasive OP-N sampling technique.

## Methods

The study is reported according to the STARD 2015 guidelines: an updated list of essential items for reporting diagnostic accuracy studies [7].

### Study design and population

This large cross-sectional diagnostic test accuracy study was embedded within the Dutch public testing infrastructure. Public testing in the Netherlands is free-of-charge but only available for government-approved test indications. At the time of the study (12 April to 14 June 2021), these indications included having symptoms of suspected SARS-CoV-2 infection or having been identified as a close contact of a SARS-CoV-2 index case via traditional contact-tracing or the contact-tracing app regardless of symptomatology at the time of notification. Participants were recruited consecutively at three Dutch public health service COVID-19 test sites across the country, located in the West-Brabant region (Breda, using the BD-Veritor Ag-RDT), in the Rotterdam-Rijnmond region (Rotterdam The Hague Airport and Ahoy, using the SD-Biosensor Ag-RDT), and in the IJsselland region (Zwolle, using the PanBio Ag-RDT). Individuals were considered eligible if they were aged 16 years or older and willing and able to sign an informed consent in Dutch.

The Dutch COVID-19 vaccination programme started on 6 January 2021. At the time of the study, an estimated 20% (12 April) to 62% (14 June) of Dutch inhabitants aged 18 or older had received at least one vaccination, ranging over time from 4–13% for 18–25-year-olds to 85–91% for 81–90-year-olds [8]. The study was conducted before the SARS-CoV-2 Delta variant became the dominant variant in the Netherlands in June 2021 (the prevalence of the Delta variant was around 8.6% in the last week of inclusions) [9, 10].

### Inclusion procedure

Participants arrived at the test sites by car or bicycle (Breda) or on foot (Rotterdam and Zwolle). Test site staff verbally verified study eligibility. Eligible individuals were given a study flyer and a participant information letter to read, after which they could indicate to site staff if they wanted to participate. After signing the informed consent form, participants completed a short questionnaire on indication for testing, presence, type, and onset of symptoms; previous SARS-CoV-2 infections; and COVID-19 vaccination status (Additional file 1: Suppl. Material 1 [6, 11–18]) while waiting for sampling.

### Specimen collection and testing

A trained test site staff member took two swabs from each study participant: one for molecular reference testing and the other for the Ag-RDT. The molecular reference test was performed in a centralised laboratory in each region, whereas the Ag-RDT was performed at the test sites. In the first phase of the study, the Ag-RDT swabs were collected using the sampling method that was routinely used at the test site, i.e. deep NP for SD-Biosensor and PanBio, and superficial OP-N (about 2.5 cm deep) for BD-Veritor. In the second phase of the study, we evaluated the SD-Biosensor and PanBio tests using superficial OP-N sampling. Ag-RDTs were conducted and interpreted in accordance with the manufacturer’s instructions; results of the BD-Veritor Ag-RDT were determined visually instead of using a Veritor Plus Analyzer [11].

While molecular testing was used as the reference standard in all three centralised laboratories, the sampling and molecular testing details varied slightly (Additional file 1: Suppl. Material 2). In Breda and Zwolle, OP-N sampling was combined with RT-PCR or transcription-mediated amplification (TMA) testing, respectively. Samples that tested positive by TMA in Zwolle were subsequently tested by RT-PCR to generate a Ct value. The Rotterdam site used combined oropharyngeal and nasopharyngeal (OP-NP) sampling combined with RT-PCR. The platforms used were Roche cobas 6800/8800 (Rotterdam and Breda, respectively) and ABI-7500 (Zwolle) for RT-PCR and the Hologic Panther system (Aptima SARS-CoV2 assay) for TMA (Additional file 1: Suppl. Material 2).

All staff assessing test results were blinded to the results of the other test. In the first phase of the study, the Ag-RDTs were conducted in accordance with routine test site procedures; participants were therefore informed about the Ag-RDT result but not the subsequent molecular test result. In the second phase of the study, the Ag-RDTs were not conducted according to routine practice and participants were therefore informed about the molecular test result.

In discordant cases (Ag-RDT-negative and RT-PCR-positive cases), whole genome sequencing (WGS) of the primary clinical sample was performed when the viral load was above a cut-off of ≥5.2 log10 SARS-CoV-2 E-gene copies/mL. This is the viral load above which 95% of people with a positive molecular test had a positive virus culture in a recent study by our group [6] (Additional file 1: Suppl. Material 2).

### Outcomes and statistical analyses

**Fig. 1:**
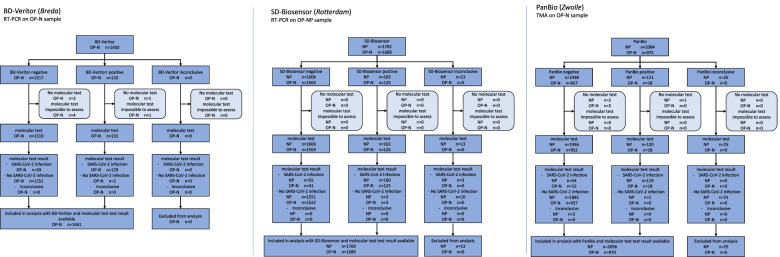
Flow of study participants. BD-Veritor BD VeritorTM System by Becton Dickinson, SD-Biosensor Roche/SD Biosensor by Roche Diagnostics, PanBio PanBio by Abbot

The primary outcomes were the diagnostic accuracies (sensitivity, specificity, positive and negative predictive values with corresponding 95% confidence intervals [CI]) of all three Ag-RDTs and sampling technique combinations, with molecular testing as the reference standard. As the number of individuals without molecular test or Ag-RDT results was very low (*n*=55 (0.7%); Fig. 1), we performed a complete case analysis.

Secondary outcomes were diagnostic accuracies above the viral load cut-off of ≥5.2 log10 SARS-CoV-2 E-gene copies/mL [6]. Additional secondary outcomes were diagnostic accuracies stratified by the presence of symptoms at time of sampling (yes or no), COVID-19 vaccination status (vaccinated with at least one dose yes or no), having had a prior SARS-CoV-2 infection (yes or no), sex (female or male), age (≥16 to ≤40 or >40 to ≤65 or >65), and testing indication (symptoms and/or close contact without symptoms). In an exploratory analysis, we performed WGS to assess whether false-negative Ag-RDT results could be linked to SARS-CoV-2 variants or specific mutations in the SARS-CoV-2 N-gene (Additional file 1: Suppl. material 2).

Finally, we used the SARS-CoV-2 test result database of the public health service test sites to identify any missed infections using pseudonymised linkage. Specifically, we determined whether participants who received a negative test result had tested positive in the subsequent 14 days by either molecular test or Ag-RDT, and analysed the interval between the initial and follow-up test. Follow-up results of participants were stratified by the results of the initial Ag-RDT and molecular reference tests and by the presence of symptoms at the time of sampling.

### Sample size calculation

Previous diagnostic accuracy studies of Ag-RDTs in people with COVID-19-like symptoms found sensitivities of around 80–85% [3–5, 11, 19]. We based our sample size calculation on an expected sensitivity of 80% for each Ag-RDT, with a margin of error of 7%, type I error of 5%, and power of 90%. We required approximately 145 positive reference tests for each Ag-RDT-molecular reference test comparison and per Ag-RDT sampling technique (routinely used versus less invasive). We expected a negligible non-response rate based on previous studies. We anticipated a SARS-CoV-2 prevalence (based on molecular testing) of 10% and closely monitored molecular test positivity rates over time in order to prolong recruitment as needed.

## Results

Between 12 April and 14 June 2021, 7980 individuals participated in the study (Fig. 1). Results for both a molecular reference test and an Ag-RDT were available for 1441 participants (99.4%) in the BD-Veritor/OP-N sampling group, 1769 participants (99.3%) in the SD-Biosensor/NP sampling group, 1689 participants (100%) in the SD-Biosensor/OP-N sampling group, 2056 participants (98.7%) in the PanBio/NP sampling group, and 970 participants (99.5%) in the PanBio/OP-N sampling group.

The SARS-CoV-2 prevalence in the Netherlands started to decline on 15 May 2021. The required number of positive reference tests had (almost) been reached in Breda and Rotterdam by then. In Zwolle, however, the second phase of the study (PanBio test using less invasive OP-N sampling) was initiated on 1 June 2021 and was terminated early on 14 June 2021 due to the low PCR test positivity percentage (only 3.4%; 33 positive molecular reference tests in 970 participants). Results of this incomplete evaluation are presented in Additional file 1: Suppl. Tables S1 and S2 and are not described any further in this manuscript.

The demographic characteristics of the study groups were similar: the mean ages ranged from 37.5 (SD 14.8) to 41.1 years (SD 16.3) and the percentages of female participants were between 50.7 and 55.6% (Table 1).

**Table 1:** Baseline characteristics of the study population, stratified by type of rapid antigen test and sampling method

Test	BD-Veritor	SD-Biosensor		PanBio
Method of sampling	Routinely used: OP-N	Routinely used: NP	Less invasive: OP-N	Routinely used: NP
Inclusion period	12–30 Apr 2021	14–20 Apr 2021	3–17 May 2021	12–22 Apr 2021
Sample size	*N* = 1441	*N* = 1769	*N* = 1689	*N* = 2056
Age [years], mean (SD)^a^	41.1 (16.3)	39.5 (15.5)	37.5 (14.8)	37.6 (14.8)
Sex, female *n* (%)^b^	798 (55.6)	894 (50.7)	856 (50.8)	1075 (52.4)
Testing indication, *n* (%)^c^				
Symptomatic	501 (34.8)	952 (53.8)	759 (44.9)	1273 (61.9)
Pre-/asymptomatic close contact of confirmed SARS-CoV-2-infected individual	800 (55.6)	688 (38.9)	752 (43.9)	594 (28.9)
Others	73 (5.1)	92 (5.2)	93 (5.5)	91 (4.4)
Unknown	67 (4.6)	37 (2.1)	85 (5.0)	98 (4.8)
Vaccinated with at least one dose, *n* (%)^d^	152 (10.5)	96 (5.4)	224 (13.3)	167 (8.1)
Type of vaccine, *n* (%)^e^				
Astra Zeneca	77 (50.7)	48 (50.0)	67 (29.9)	113 (67.7)
Janssen			7 (3.1)	
Moderna	7 (4.6)	5 (5.2)	19 (8.5)	9 (5.4)
Pfizer	63 (41.4)	36 (37.5)	121 (54.0)	43 (25.7)
Unknown	5 (3.3)	7 (7.3)	10 (4.5)	2 (1.2)
Number of vaccinations received, *n* (%)^e^				
1	107 (70.4)	75 (78.1)	169 (75.4)	136 (81.4)
2	31 (20.4)	11 (11.5)	33 (14.7)	20 (12.0)
Unknown	14 (9.2)	10 (10.4)	22 (9.8)	11 (6.6)
At least one prior SARS-CoV-2 infection, *n* (%)^f^	102 (7.1)	187 (10.6)	196 (11.6)	134 (6.5)
Symptoms at time of sampling, *n* (%)	662 (47.2)	1091 (62.4)	900 (55.0)	1470 (74.2)
Symptom onset, *n* (%)^g^				
At day of sampling	19 (2.9)	91 (8.3)	70 (7.8)	240 (16.3)
A day before sampling	189 (28.5)	482 (44.2)	374 (41.6)	610 (41.5)
Two days before sampling	218 (32.9)	282 (25.8)	209 (23.2)	332 (22.6)
Three or more days before sampling	252 (38.1)	250 (22.9)	247 (27.4)	286 (19.5)
Unknown	15 (2.3)	15 (1.4)	19 (2.1)	20 (1.4)
Type of symptoms (self-reported), *n* (%)^g,h^				
Common cold	570 (86.1)	948 (86.9)	768 (85.3)	1349 (91.8)
Shortness of breath	113 (17.1)	137 (12.6)	121 (13.4)	197 (13.4)
Fever	72 (10.9)	146 (13.4)	126 (14.0)	157 (10.7)
Coughing	308 (46.5)	450 (41.2)	342 (38.0)	584 (39.7)
Loss of taste or smell	24 (3.6)	43 (3.9)	41 (4.6)	55 (3.7)
Muscle ache	88 (13.3)	137 (12.6)	100 (11.1)	143 (9.7)
Other symptoms	37 (5.6)	18 (1.6)	54 (6.0)	74 (5.0)

In the Netherlands, individuals are notified of a close contact by the Dutch public health service test-and-trace programme and/or the Dutch contact-tracing mobile phone application (the CoronaMelder app) and/or an individual with a confirmed SARS-CoV-2 infection (index case)

*NP* deep nasopharyngeal, *OP-N* combined oropharyngeal and nasal sampling, *SD* standard deviation

^a^Age was not available from 3, 4, 4, and 2 participants in the BD-Veritor group, SD-Biosensor NP group, SD-Biosensor OP-N group, and PanBio NP group, respectively

^b^Sex was not available from 6, 4, 3, and 4 participants in the BD-Veritor group, SD-Biosensor NP group, SD-Biosensor OP-N group, and PanBio NP group, respectively

^c^Indication for testing was referral for other reasons for 73, 92, 93, and 91 participants and unknown for 67, 37, 85, and 98 participants in the BD-Veritor group, SD-Biosensor NP group, SD-Biosensor OP-N group, and PanBio NP group, respectively

^d^COVID-19 vaccination status was not available from 34, 14, 53, and 72 participants, including 7, 0, 4, and 7 with a positive molecular test result, in the BD-Veritor group, SD-Biosensor NP group, SD-Biosensor OP-N group, and PanBio NP group, respectively

^e^Percentage calculated as the proportion of those vaccinated

^f^Previous SARS-CoV-2 infection information was not available from 48, 14, 56, and 72 participants in the BD-Veritor group, SD-Biosensor NP group, SD-Biosensor OP-N group, and PanBio NP group, respectively

^g^Percentage calculated as the proportion of those with symptoms at the time of sampling

^h^Totals add up to a number higher than the number of individuals with symptoms at the time of sampling because individuals could report more than one symptom

**Fig. 2:**
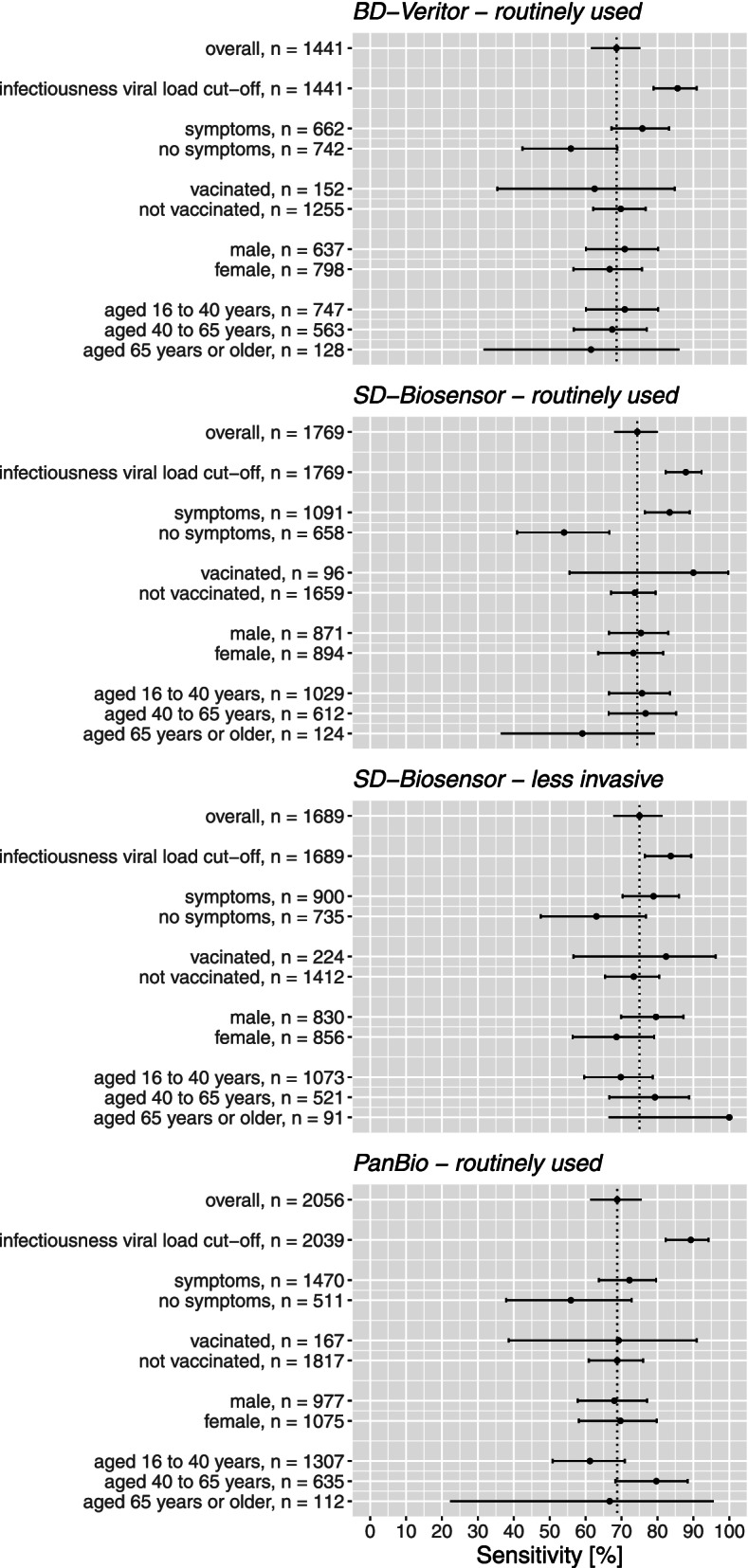
Sensitivities with 95% confidence intervals of the various antigen rapid test-molecular reference standard test comparisons, stratified according to symptomatology, COVID-19 vaccination status, sex, and age. BD-Veritor BD Veritor^TM^ System by Becton Dickinson, SD-Biosensor Roche/SD Biosensor by Roche Diagnostics, PanBio PanBio by Abbot

Table 2 shows the results of the primary analysis, the secondary analyses restricted to samples with a viral load above the cut-off, and the secondary analyses stratified by the presence or absence of symptoms at the time of sampling. Secondary analyses stratified by COVID-19 vaccination status, sex, and age are presented in Additional file 1: Suppl. Table S3. Sensitivities of all primary and secondary analyses are visualised in Fig. 2. Additional file 1: Suppl. Tables S4 to S8 show 2×2 tables for each Ag-RDT-sampling technique combination.

**Table 2:** Diagnostic accuracy variables of three rapid antigen tests, with different sampling methods. Values are percentages (95% confidence interval) unless stated otherwise

Analysis	Sampling method	No.	Prevalence^**a**^ (%)	Sensitivity	Specificity	PPV	NPV
**BD-Veritor System (Beckton Dickinson)**							
Primary analysis	OP-N	1441	13.0	68.6 (61.5 to 75.2)	99.8 (99.4 to 100.0)	98.5 (94.6 to 99.8)	95.5 (94.2 to 96.6)
Secondary (stratified) analysis							
Viral load above the cut-off^c^	OP-N	1441	10.1	85.6 (78.9 to 90.9)	99.5 (99.0 to 99.8)	95.4 (90.3 to 98.3)	98.4 (97.6 to 99.0)
Symptoms present at sampling^b^							
Yes	OP-N	662	18.1	75.8 (67.2 to 83.2)	99.8 (99.0 to 100.0)	98.9 (94.1 to 100.0)	94.9 (92.8 to 96.6)
No	OP-N	742	8.0	55.9 (42.4 to 68.8)	99.9 (99.2 to 100.0)	97.1 (84.7 to 99.9)	96.3 (94.7 to 97.6)
**SD-Biosensor (Roche Diagnostics)**							
Primary analysis	NP	1769	12.2	74.4 (68.0 to 80.1)	99.8 (99.4 to 100.0)	98.2 (94.7 to 99.6)	96.6 (95.6 to 97.4)
	OP-N	1689	9.7	75.0 (67.7 to 81.4)	99.8 (99.4 to 100.0)	97.6 (93.2 to 99.5)	97.4 (96.5 to 98.1)
Secondary (stratified) analysis							
Viral load above the cut-off^c^	NP	1769	10.3	87.9 (82.3 to 92.3)	99.8 (99.4 to 100.0)	98.2 (94.7 to 99.6)	98.6 (97.9 to 99.1)
	OP-N	1689	8.3	83.7 (76.5 to 89.4)	99.5 (99.0 to 99.8)	93.7 (87.9 to 97.2)	98.5 (97.8 to 99.1)
Symptoms present at sampling^b^							
Yes	NP	1091	13.8	83.4 (76.5 to 89.0)	99.8 (99.2 to 100.0)	98.4 (94.5 to 99.8)	97.4 (96.2 to 98.3)
	OP-N	900	12.7	78.9 (70.3 to 86.0)	99.7 (99.1 to 100.0)	97.8 (92.4 to 99.7)	97.0 (95.6 to 98.1)
No	NP	658	9.6	54.0 (40.9 to 66.6)	99.8 (99.1 to 100.0)	97.1 (85.1 to 99.9)	95.3 (93.4 to 96.9)
	OP-N	735	6.3	63.0 (47.5 to 76.8)	100.0 (99.5 to 100.0)	100.0 (88.1 to 100.0)	97.6 (96.2 to 98.6)
**PanBio (Abbott)**							
Primary analysis	NP	2056	8.4	68.8 (61.3 to 75.6)	99.9 (99.7 to 100.0)	99.2 (95.4 to 100.0)	97.2 (96.4 to 97.9)
Secondary (stratified) analysis:							
Viral load above the cut-off^cd^	NP	2039	5.9	89.3 (82.3 to 94.2)	99.9 (99.6 to 100.0)	98.2 (93.6 to 99.8)	99.3 (98.9 to 99.6)
Symptoms present at sampling^b^							
Yes	NP	1470	9.0	72.2 (63.7 to 79.6)	99.9 (99.6 to 100.0)	99.0 (94.4 to 100.0)	97.3 (96.3 to 98.1)
No	NP	511	6.7	55.9 (37.9 to 72.8)	100.0 (99.2 to 100.0)	100.0 (82.4 to 100.0)	97.0 (95.0 to 98.3)

*NC* not calculated because all Ag-RDT results were negative, *NP* deep nasopharyngeal, *OP-N* combined oropharyngeal and nasal sampling, *PPV* positive predictive value, *NPV* negative predictive value

^a^SARS-CoV-2 infection based on the molecular test result

^b^Symptoms not available for 37, 20, 53, and 75 participants, including 9, 1, 4, and 7 with a positive molecular test result, in the BD-Veritor group, SD-Biosensor NP group, SD-Biosensor OP-N group, and PanBio NP group, respectively

^c^The viral load cut-off was 5.2 log10 SARS-CoV-2 E-gene copies/mL. This was the viral load above which 95% of people with a positive RT-PCR test result had a positive viral culture in a recent study by our group [6]

^d^Viral load unavailable for 17 participants in the PanBio NP group

### Routinely used Ag-RDT sampling method

SARS-CoV-2 prevalence (by molecular reference test) was 13.0% (188/1441) in the BD-Veritor group, 12.2% (215/1769) in the SD-Biosensor group, and 8.4% (173/2056) in the PanBio group. Overall sensitivities were 68.6% [61.5–75.2%] for BD-Veritor, 74.4% [68.0–80.1%] for SD-Biosensor, and 68.8% [61.3–75.6%] for PanBio (Table 2, Fig. 2).

Among those with a positive molecular test result, the percentage of participants with a viral load above the cut-off was 77.7% (146/188) in the BD-Veritor group, 85.1% (183/215) in the SD-Biosensor group, and 70.0% (121/173) in the PanBio group. Using this viral load cut-off, the sensitivities were 85.6% [78.9–90.9%] for BD-Veritor, 87.9% [82.3–92.3%] for SD-Biosensor, and 89.3% [82.3–94.2%] for PanBio.

Sensitivities ranged from 72.2 to 83.4% in individuals who were symptomatic at the time of sampling and from 54.0 to 55.9% in those who were asymptomatic (Table 2, Fig. 2). We found no evidence of a differential impact on diagnostic accuracy by COVID-19 vaccination status, sex, and age (Fig. 2, Additional file 1: Suppl. Table S3).

Specificities were >99%, and positive and negative predictive values were >95%, for all three Ag-RDT in most analyses (Table 2 and Additional file 1: Suppl. Table S3).

### Less invasive OP-N sampling method combined with SD-Biosensor

The SARS-CoV-2 prevalence (by molecular reference test) was 9.7% (164/1689) and the sensitivity was 75.0% [67.7–81.4%] (Table 2, Fig. 2).

Among those with a positive molecular test result, the percentage of participants with a viral load above the cut-off was 86.0% (141/164). Using this viral load cut-off, the sensitivity was 83.7% [76.5–89.4%].

Sensitivities were 78.9% in symptomatic individuals and 63.0% in those who were asymptomatic at the time of sampling (Table 2, Fig. 2). We found no evidence of a differential impact on diagnostic accuracy by COVID-19 vaccination status, sex, and age (Additional file 1: Suppl. Table S3).

Specificities were >99%, and positive and negative predictive values were >95%, in most analyses (Table 2 and Additional file 1: Suppl. Table S3).

### Follow-up to identify missed infections

All but six of all study participants could be linked with the national test results database of the public health services for the follow-up analyses.

In the first phase of the study, participants received the result of the Ag-RDT test and not the result of the molecular reference test. This Ag-RDT result was false-negative in 3.5% of participants (Table 3). Of the participants who tested Ag-RDT-negative in the study, 18.3% had another SARS-CoV-2 test done at a public health service testing site within 14 days, and 4.4% tested positive on that repeat test (Table 3). These percentages were much higher for those with a false-negative than a true-negative Ag-RDT result during the study: 61.3% versus 16.8% (*χ*^2^ =215, *p*<.001) for having a repeat test and 55.4% versus 2.6% (*χ*^2^ =1076, *p*<.001) for that repeat test being positive (Table 3). Furthermore, the interval between the Ag-RDT-negative result during the study and the subsequent positive test results within the 14-day follow-up period was shorter for participants with false-negative results during the study (median 3 days, interquartile range (IQR) 3 days) than for those with true-negative results (median 5 days, IQR 3 days; *U*=2810, *p*<.001). Finally, being asymptomatic at the time of initial testing was associated with a higher likelihood of testing positive during the 14-day follow-up period for both false-negative and true-negative individuals (Table 3).

**Table 3:** Follow-up of participants who initially received a negative test result

	Phase 1 (Ag-RDT result communicated)	Phase 2 (molecular test result communicated)
Initial test result negative, *n*	4847	2461
*Initial test result false negative, n (%)*	*168 (3.5)*	*n/a*^a^
*Initial test result true negative, n (%)*	*4697 (96.5)*	*n/a*^a^
At least one subsequent SARS-CoV-2 test registered within 14 days^b^, *n* (%)	887 (18.3)	284 (11.5)
*Initial test result false negative, n (%)*	*103 (61.3)*	*n/a*^a^
*Initial test result true negative, n (%)*	*784 (16.8)*	*n/a*^a^
SARS-CoV-2-positive test within 14 days, *n* (%)	213 (4.4)	28 (1.1)
*Initial test result false negative, n (%)*	*93 (55.4)*	*n/a*^a^
*Initial test result true negative, n (%)*	*120 (2.6)*	*n/a*^a^
**Stratified analysis according to symptomology at time of initial testing**		
Initial test result negative, *n*	4715^c^	2392^d^
*Symptomatic, n (%)*	*2893 (61.4)*	*1427 (59.7)*
*Asymptomatic, n (%)*	*1822 (38.6)*	*965 (40.3)*
Initial test result false negative, *n*	161	n/a^a^
*Symptomatic, n (%)*	*91 (56.5)*	*n/a*^a^
*Asymptomatic, n (%)*	*70 (43.5)*	*n/a*^a^
Initial test result true negative, *n*	4554	n/a^a^
*Symptomatic, n (%)*	*2802 (61.5)*	*n/a*^a^
*Asymptomatic, n (%)*	*1752 (38.5)*	*n/a*^a^
SARS-CoV-2-positive test within 14 days		
Initial test result negative	*-*	-
*Symptomatic, n (%)*	*87 (3.0)*	*12 (0.8)* ^*^
*Asymptomatic, n (%)*	*114 (6.3)*	*16 (1.7)* ^*^
Initial test result false negative	*-*	
*Symptomatic, n (%)*	42 (46.2)**	*n/a*^a^
*Asymptomatic, n (%)*	44 (62.9)**	*n/a*^a^
Initial test result true negative	-	
*Symptomatic, n (%)*	45 (1.6)***	*n/a*^a^
*Asymptomatic, n (%)*	70 (4.0)***	*n/a*^a^

*Ag-RDT* rapid lateral flow antigen diagnostic tests, *n/a* not applicable

^a^Not applicable since the molecular reference test result was communicated in phase 2

^b^Based on pseudonymised linkage to SARS-CoV-2 test results database of the public health service test sites

^c^Symptom status not available for 132 participants

^d^Symptom status not available for 69 participants

^*^χ^2^ =3.3, *p*=.068

^**^χ^2^ =4.4, *p*=.035

^***^χ^2^ =25, *p*<.001

In the second phase of the study, participants received the molecular reference test result and not the Ag-RDT test result. Of the participants who tested reference test negative in the study, 11.5% had another SARS-CoV-2 test done at a public health service testing site within 14 days, and 1.1% tested positive on that repeat test (Table 3). The median interval between the initial negative reference test and a positive follow-up test was 6 days (IQR 5 days). Participants who were asymptomatic at the time of initial testing were more likely to test positive during the 14 day follow-up period than those who were symptomatic, but the difference was not statistically significant (Table 3).

## Discussion

The BD-Veritor, SD-Biosensor, and PanBio lateral flow Ag-RDTs are the three most used SARS-CoV-2 point-of-care tests in the Netherlands. They underwent limited diagnostic accuracy evaluations prior to their approval for use in the public testing programme but were never evaluated in a large community-based study with nationwide reach. In addition, the public’s desire to move away from deep NP sampling increased over time, but the diagnostic accuracies of the SD-Biosensor and PanBio tests using OP-N sampling were not yet known.

Our study found that the three Ag-RDTs combined with their routine sampling techniques had sensitivities of 68.6 to 74.4%, increasing to at least 85.6% after a viral load cut-off was applied. Sensitivities ranged from 72.2 to 83.4% in individuals who were symptomatic at the time of sampling and from 54.0 to 55.9% in those who were asymptomatic. We found no evidence of a differential impact on the diagnostic accuracy of COVID-19 vaccination status, sex, and age. For SD-Biosensor, the less invasive OP-N sampling technique yielded similar sensitivities in the primary and secondary stratified analyses as the deep NP approach. Specificities and positive and negative predictive values were high for all Ag-RDT sampling technique combinations.

Follow-up analyses of persons with false-negative Ag-RDT results show that more than 55% (symptomatic vs asymptomatic at time of initial sampling: 46% vs 63%) tested positive in the 14 days after the initial test, whereas positive test results within 14 days after initial testing occurred in 1.1% of people with a negative initial molecular test.

### Comparison with other studies

The overall unstratified sensitivities of the Ag-RDTs in our study were substantially lower than those reported in Ag-RDT evaluations performed earlier in the pandemic in the Netherlands [11, 19]. We hypothesise that the reason for this is because only symptomatic individuals could access SARS-CoV-2 testing in the Netherlands until 1 December 2020. The sensitivities in our study for individuals who were symptomatic at the time of sampling were indeed similar to those in the earlier evaluation studies. The sensitivities in our study for individuals who were asymptomatic at the time of sampling are in line with those that we found in our recent study in asymptomatic and presymptomatic close contacts [6]. The positivity percentages during the 14-day follow-up period of people with a negative initial molecular test in our study was 1.1%, which was slightly lower than the 1.7% found in our previous study among close contacts. This can likely be attributed to the fact that close contacts are at higher risk of testing SARS-CoV-2-positive than those testing for other reasons [6].

To our knowledge, a direct comparison of the diagnostic accuracy of any Ag-RDT combined with superficial OP-N versus deep NP sampling has not been conducted. The superficial OP-N sampling technique is currently routinely used in the Netherlands for the BD-Veritor Ag-RDT. The sensitivities that we observed for this combination were similar to those of the other two Ag-RDTs combined with deep NP sampling, as well as the SD-Biosensor Ag-RDT combined with OP-N sampling. The use of more convenient sampling methods holds potential for reducing the threshold for SARS-CoV-2 testing.

### Strengths and limitations of this study

Strengths include the protocolised nature of the study, the large sample size covering multiple test sites nationwide, the high data completeness, collection of samples for the index and reference tests at the same time, the implementation of index and reference tests by trained staff who were blinded to the result of the other test, and the availability of follow-up information for participants who received negative test results.

Our study also has some limitations. First, our study does not provide a direct comparison of the diagnostic accuracy of the various Ag-RDTs or sampling techniques. However, we did use similar eligibility criteria and study protocols across the three study sites, and we included participants at the three study sites during the same time period. Second, the reference standards that we used were molecular tests, but platforms and test kits used differed among the three centralised laboratories (Rotterdam: OP-NP sampling with RT-PCR (Roche cobas 6800), Breda: OP-N sampling with RT-PCR (Roche cobas 8800), Zwolle: OP-N sampling with TMA (Hologic Panther system) with TMA-positive samples tested by RT-PCR (ABI-7500)). However, the diagnostic accuracies of all molecular tests used are similarly high [20, 21], and we therefore believe that this has not influenced our findings significantly. In addition, Ct values determined by the different platforms were comparable (Additional file 1: Suppl. Material 2). Third, we were unable to meet the predefined sample size in the PanBio/OP-N sampling group. These results are therefore not sufficiently robust and should be interpreted with great caution. Fourth, while the Omicron variant is the dominant SARS-CoV-2 variant in the Netherlands at time of writing, this variant was not present during our study period and the estimated prevalence of Delta was 8.6% during the last week of inclusions. We checked whether false-negative Ag-RDT results could be linked to specific virus lineages by WGS and we did not find a signal confirming this hypothesis. Fifth, we applied a viral load cut-off above which 95% of RT-PCR test positive individuals in our previous study was virus culture positive [6]. The previous study population, however, consisted of almost completely unvaccinated participants, whereas the proportion of vaccinated individuals in the present study reached 20% at the end of the study. Whether this would have impacted the applied viral load cut-off is unknown. Also, the applied viral load cut-off should be interpreted with some caution. Albeit evidence is accumulating that both infectivity in culture and viral load, and viral load and secondary attack rate, are correlated [22–24], the exact viral load cut-off below which no transmissions take place is still unknown. Missed infections below the applied viral load cut-off can therefore not be discounted as of limited relevance, especially because viral loads may alter over time during the infection. Sixth, our sample size calculation was based on the primary analysis and the diagnostic accuracy parameters are therefore less precise for the secondary stratified analyses. For example, a differential impact of COVID-19 vaccination status on the diagnostic accuracy of Ag-RDTs might be anticipated given that vaccinated individuals have shorter periods of high viral loads and lower virus viability by Ct values than unvaccinated individuals, resulting in lower transmissibility [24–28]. Further studies on the potential impact of COVID-19 vaccination on Ag-RDT diagnostic accuracies are warranted. Finally, we did not actively follow-up participants who had received a negative test result but collected follow-up information from the public health services test result database through pseudonymised linkage. Active follow-up, including repeat testing in all study participants, would have reduced the uncertainty around false-negative Ag-RDT results completely, as was also recommended in a recent guidance paper [29]. Unfortunately, we could not implement this because of ethical and logistic constraints, as our study was embedded in busy public health service test sites. Also, we cannot be certain that all positive tests within the maximum incubation period after a negative initial test represent false-negative tests; they could also have resulted from a new SARS-CoV-2 exposure after the initial test. However, this is true for individuals with a negative Ag-RDT and for those with a negative molecular test at inclusion, and the difference in positivity percentages during follow-up was strikingly large.

### Policy implications

As the COVID-19 pandemic will gradually move into a different phase with less emphasis on finding all infections, public health policies may want to rely more on Ag-RDTs instead of molecular testing because of simplified logistics and reduced delays. The more frequent use of Ag-RDTs instead of molecular testing will inevitably lead to an increase in the number of missed infections, especially when used in asymptomatic individuals, e.g. for travelling and/or access to events. We observed that about 60% of persons with false-negative Ag-RDT results returned for testing, and 55% tested positive, within 14 days after the initial negative test with a median delay of 3 days. About half of them did not have symptoms at the time of initial sampling, and those participants were more likely to have a positive follow-up test (symptomatic vs asymptomatic at time of initial sampling: 46% vs 63%). This suggests that a considerable portion of individuals adhered to the advice to return for testing if symptoms develop or worsen after a negative test and also illustrates the importance of emphasizing the need for re-testing to individuals with a negative Ag-RDT should such events occur. However, as over half of all infections are estimated to occur before the onset of symptoms, these missed early infections do pose a relevant risk for further transmission [30, 31]. Furthermore, the 45% of individuals with a false-negative Ag-RDT who are never diagnosed pose an even greater risk of onward transmission. The extent to which the advantages of Ag-RDTs outweigh their lower sensitivities depends on several aspects, including the potential consequences of missed infections.

Furthermore, as the prevalence of SARS-CoV-2 declines, the positive predictive value of Ag-RDT results will decrease, meaning a larger proportion of positive test results will be false positive [3]. In such circumstances, the risk of false-positive results with Ag-RDTs could be mitigated by confirmatory molecular testing.

## Conclusions

Compared to molecular testing, the sensitivities of three widely used SARS-CoV-2 Ag-RDTs when applying the routinely used sampling techniques were at least 69% and increased to at least 85% after a viral load cut-off was applied. Sensitivities ranged between 54 and 56% in those who were asymptomatic at the time of sampling, meaning that around half of infections might be missed in this population. Follow-up analyses revealed that over 55% of asymptomatic persons with a false-negative Ag-RDT result tested positive within 14 days after the initial test emphasizing the need for re-testing should symptoms develop and education of the public about a high potential of false-negative Ag-RDTs when asymptomatic. In high-risk situations, such as testing of vulnerable people in care facilities, severely ill patients, or healthcare workers, molecular testing remains the preferred option. For SD-Biosensor, the less invasive OP-N sampling technique yielded similar diagnostic accuracies for Ag-RDT-molecular test comparisons as the deep NP approach. Adopting this more convenient sampling method might reduce the threshold for professional SARS-CoV-2 testing.

## Supplementary Information

**Additional file 1 **: **Suppl. Table S1.** Baseline characteristics of the study population included in the PanBio test with less invasive sampling technique evaluation. **Suppl. Table S2.** Diagnostic accuracy variables of PanBio-molecular reference standard test comparison with less invasive sampling. Values are percentages (95% confidence interval) unless stated otherwise. **Suppl. Table S3.** Diagnostic accuracy variables of additional secondary analyses of three rapid antigen tests, with different sampling methods. Values are percentages (95% confidence interval) unless stated otherwise. **Suppl. Table S4.** Two-by-two tables used in primary and secondary analysis to determine diagnostic accuracy parameters of the BD Veritor^TM^ System by Becton Dickinson (‘BD-Veritor’) using routine sampling (OP-N). **Suppl. Table S5.** Two-by-two tables used in primary and secondary analysis to determine diagnostic accuracy parameters of the Roche/SD Biosensor by Roche Diagnostics (‘SD-Biosensor’) using routine sampling (NP)*.* **Suppl. Table S6.** Two-by-two tables used in primary and secondary analysis to determine diagnostic accuracy parameters of the Roche/SD Biosensor by Roche Diagnostics (‘SD-Biosensor’) using less invasive sampling (OP-N). **Suppl. Table S7.** Two-by-two tables used in primary and secondary analysis to determine diagnostic accuracy parameters of the PanBio by Abbot (‘PanBio’) using routine sampling (NP). **Suppl. Table S8.** Two-by-two tables used in primary and secondary analysis to determine diagnostic accuracy parameters of the PanBio by Abbot (‘PanBio’) using less invasive sampling (OP-N). **Suppl. Table S9.** GISAID data contributors acknowledgement table. **Suppl. Material 1.** Short questionnaire (translated from Dutch). **Suppl. Material 2.** Specimen collection, SARS-CoV-2 diagnostic testing, and SARS-CoV-2 virus culture procedures.

## Data Availability

Individual participant data collected during the study will be available, after deidentification of all participants. Data will be available to researchers who provide a methodologically sound proposal to achieve the aims in the approved proposal. Proposals should be directed to the corresponding author to gain access to the data. Data requestors will need to sign a data sharing agreement. The corresponding author (RPV, the manuscript’s guarantor) affirms that the manuscript is an honest, accurate, and transparent account of the study being reported; that no important aspects of the study have been omitted; and that any discrepancies from the study as originally planned (and, if relevant, registered) have been explained. The study protocol is available upon request by contacting Roderick Venekamp at r.p.venekamp@umcutrecht.nl.

## Abbreviations

Ag-RDTs: Rapid lateral flow antigen diagnostic tests
BD-Veritor: BD Veritortm System by Becton Dickinson
CI: Confidence interval
COVID-19: Coronavirus disease 2019
IQR: Interquartile range
NP: Deep nasopharyngeal
OP-N: Oropharyngeal combined with superficial nasal
OP-NP: Combined oropharyngeal and nasopharyngeal
PanBio: PanBio by Abbott
RT-PCR: Real-time reverse-transcriptase polymerase chain reaction
SARS-CoV-2: Severe acute respiratory syndrome coronavirus-2
SD-Biosensor: Roche/SD Biosensor by Roche Diagnostics
STARD 2015: Standards for Reporting of Diagnostic Accuracy Studies 2015
TMA: Transcription-mediated amplification
WGS: Whole genome sequencing
